# Inverse association between periumbilical fat and longevity mediated by complement C3 and cardiac structure

**DOI:** 10.18632/aging.104113

**Published:** 2020-11-18

**Authors:** Shihui Fu, Yao Yao, Shengzheng Wu, Juelin Deng, Faqin Lv, Yali Zhao

**Affiliations:** 1 Department of Geriatric Cardiology, Chinese People's Liberation Army General Hospital, Beijing 100853, China; 2 Department of Cardiology, Hainan Hospital of Chinese People's Liberation Army General Hospital, Sanya 572013, China; 3 Center for the Study of Aging and Human Development and Geriatrics Division, Medical School of Duke University, Durham, NC 27708, USA; 4 Center for Healthy Aging and Development Studies, National School of Development, Peking University, Beijing 100871, China; 5 Department of Ultrasound, Hainan Hospital of Chinese People's Liberation Army General Hospital, Sanya 572013, China; 6 Central Laboratory, Hainan Hospital of Chinese People's Liberation Army General Hospital, Sanya 572013, China

**Keywords:** cardiac structure, longevity, complement C3, periumbilical fat, abdominal obesity

## Abstract

Although abdominal obesity plays a fundamental role in the onset of immune and inflammatory reactions leading to cardiac abnormalities and premature mortality, the potential association between periumbilical fat and longevity mediated by the antibody-complement system and/or cardiac structure and function remains unclear. To address this issue, we collected biochemical and morphological data from 419 centenarians and 491 non-centenarian oldest-old individuals from the China Hainan Centenarian Cohort Study. Centenarians had lower waist circumference (WC), periumbilical fat thickness (PFT), serum complement C3 level, right atrium end-systolic diameter (RAESD), left atrium end-systolic diameter (LAESD), and left ventricular end-diastolic diameter (LVEDD) than non-centenarians (P<0.05 for all comparisons). WC, PFT, complement C3 levels, RAESD, LAESD, and LVEDD were inversely associated with centenarians (P<0.05 for all variables). Complement C3 level, LAESD, and LVEDD were positively associated with PFT and WC (P<0.05 for all variables). RAESD was positively associated with WC and complement C3 level (P<0.05 for both variables). Centenarians had less periumbilical fat, a weaker complement system, and smaller cardiac structure than non-centenarians. Importantly, periumbilical fat was inversely associated with longevity mediated by complement C3 and cardiac structure. This study suggests that successful aging can be promoted by increased efforts to prevent abdominal obesity.

## INTRODUCTION

With a rapidly increasing prevalence worldwide, abdominal obesity has become a significant public health problem [1–5]. Compared to subcutaneous fat, abdominal fat is more pro-inflammatory; it was shown for instance that the synthesis of components of the complement system, a key mediator of inflammatory responses and innate immunity, is exacerbated in abdominal fat depots of obese individuals [6, 7]. In turn, excessive accumulation of abdominal fat is linked to the development of metabolic syndrome and a range of chronic health conditions including diabetes, cardiovascular disease, and cancer. Not surprisingly, studies have shown that obesity is associated with reduced life expectancy [8, 9]. As a hallmark of human aging, the accrual of abdominal fat is likely to have, also in non-obese people, a significant impact on longevity. Periumbilical fat is measured by noninvasive ultrasound and represents a useful tool in determining abdominal obesity. Whereas limited data on the association between periumbilical fat and antibody and complement systems are currently available from studies carried out in the United States and European countries, studies addressing these aspects in Chinese oldest-old population are still lacking [10, 11]. Asians are predisposed to have a higher ratio of abdominal obesity to overall obesity than Westerners, suggesting that the adverse impact of abdominal obesity on longevity may be more pronounced in the Asian population [12, 13]. So far, no studies have analyzed the potential association between periumbilical fat and longevity in relation to antibody and complement systems.

Cardiac diseases are the leading cause of premature mortality worldwide [14], with abdominal obesity being a fundamental cause of abnormal cardiac structure and function [15]. Indeed, a 2010 survey conducted by China’s Ministry of Health suggested that abdominal obesity contributes to increased mortality rate and reduced life expectancy by impairing cardiac structure and function [16]. However, since racial differences in body build, fat distribution, and genetic background may determine distinct effects of periumbilical fat on cardiac structure and function, aging and longevity research conducted in Western and Asian populations may conceivably yield different results [13].

**Figure 1:**
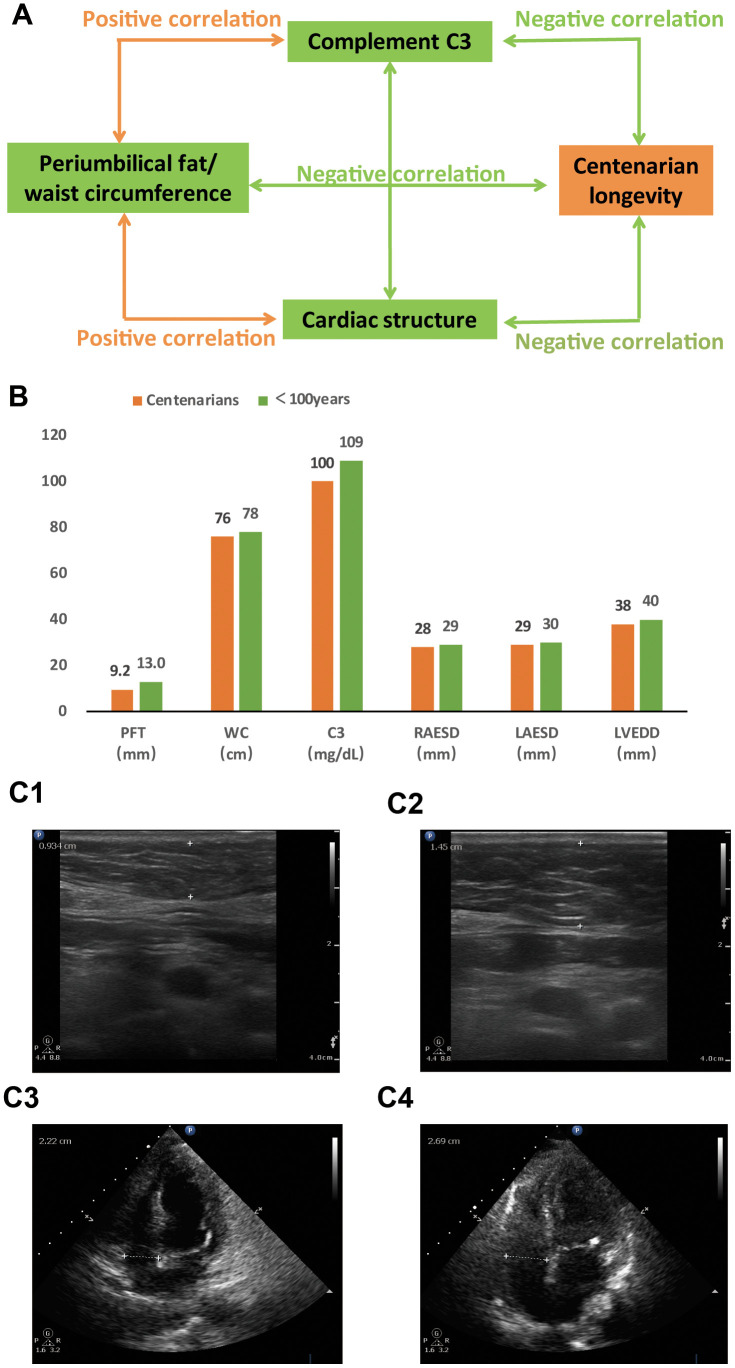
**Comparisons between centenarians and non-centenarians in Chinese oldest-old population.** (**A**) Association network between periumbilical fat and longevity mediated by antibody and complement systems and cardiac structure and function (P<0.05 for all variables). (**B**) Graphic summary of periumbilical fat, complement **C3**, and cardiac structure in centenarians and non-centenarian oldest-old population (P<0.05 for all comparisons). (**C**) Representative ultrasound images of periumbilical fat (top) and cardiac structure (bottom) from a centenarian (**C1** and **C3**) and a non-centenarian oldest-old individual (**C2** and **C4**). Abbreviations: PFT: periumbilical fat thickness; WC: waist circumference; **C3**: complement **C3**; RAESD: right atrium end-systolic diameter; LAESD: left atrium end-systolic diameter; LVEDD: left ventricular end-diastolic diameter.

Centenarians have been suggested to have delayed or escaped onset and interaction of age-related abnormalities, and are thus considered a prototype of successful aging [17, 18]. Therefore, studies analyzing associations between genetic, physical, and metabolic factors influencing centenarians will provide valuable information to formulate strategies promoting successful aging and longevity [10, 19]. To our knowledge, few studies have specifically discussed these associations in Chinese oldest-old population, including centenarians. The China Hainan Centenarian Cohort Study (CHCCS) was launched to establish a multidimensional database of longevity-related information. Based on demographic data from Hainan, the area with the highest population density of centenarians in China, the CHCCS constitutes a significant population-based sample of Chinese centenarians. Based on CHCCS data, the current study was designed to test the hypothetical association between periumbilical fat and longevity mediated by the antibody-complement systems and cardiac structure and function in a Chinese oldest-old population (Figure 1A).

## RESULTS

### Comparisons between centenarian and non-centenarian oldest-old populations

The median age of all participants was 92 years (84 years for non-centenarians, 102 years for centenarians; range: 80-116 years of age). The characteristics of the study’s 419 centenarians and 491 non-centenarians are shown in Table 1. Centenarians had lower periumbilical fat thickness (PFT), waist circumference (WC), diastolic blood pressure (DBP), total cholesterol (TC) level, low-density lipoprotein cholesterol (LDL-C) level, complement C3 level, complement C4 level, immunoglobulin M (IgM) level, right atrium end-systolic diameter (RAESD), pulmonary artery diameter (PAD), left atrium end-systolic diameter (LAESD), left ventricular end-diastolic diameter (LVEDD), interventricular septum thickness (IVST), and aorta diameter (AOD) than non-centenarians (Figure 1B, 1C; P<0.05 for all comparisons). Centenarians had higher SBP, fasting blood glucose (FBG) level, immunoglobulin G (IgG) level, immunoglobulin E (IgE) level, immunoglobulin kappa level, and right ventricle end-diastolic diameter (RVEDD) than non-centenarians (P<0.05 for all comparisons). The proportion of males was lower in the centenarians than non-centenarians (P<0.05). There was no difference in TG level, HDL-C level, immunoglobulin A (IgA) level, immunoglobulin lambda level, and left ventricular ejection fraction (LVEF) between the two groups (P>0.05 for all comparisons).

**Table 1:** Characteristics of all oldest old participants.

**Characteristic**	**Total (n=910)**	**Centenarians (n=419)**	**<100 years (n=491)**	**P**
Age (years)^*^	92(84, 102)	102(101, 104)	84(82, 88)	<0.001
Males (%)	286(31.4)	90(21.5)	196(39.9)	<0.001
Periumbilical fat thickness (cm)^*^	1.15(0.76, 1.60)	0.92(0.60, 1.40)	1.30(0.90, 1.70)	<0.001
Waist circumference (cm)^*^	76(71, 83)	76(70, 81)	78(72, 86)	<0.001
Systolic blood pressure (mmHg)^*^	149(132, 169)	151(136, 173)	146(130, 165)	0.001
Diastolic blood pressure (mmHg)^*^	77(69, 86)	76(67, 83)	79(71, 89)	<0.001
Fasting blood glucose (mmol/L)^*^	4.59(3.97, 5.48)	4.89(4.25, 5.72)	4.31(3.74, 5.20)	<0.001
Triglyceride (mmol/L)^*^	1.07(0.81, 1.48)	1.06(0.81, 1.44)	1.07(0.81, 1.51)	0.584
Total cholesterol (mmol/L)^*^	4.75(4.21, 5.51)	4.59(4.10, 5.29)	4.92(4.34, 5.74)	<0.001
Low-density lipoprotein cholesterol (mmol/L)^*^	2.83(2.36, 3.46)	2.76(2.32, 3.29)	2.94(2.43, 3.60)	<0.001
High-density lipoprotein cholesterol (mmol/L)^*^	1.39(1.15, 1.69)	1.41(1.20, 1.67)	1.37(1.13, 1.70)	0.186
Complement C3 (mg/dL)^*^	105(91, 120)	100(88, 112)	109(95, 127)	<0.001
Complement C4 (mg/dL)^*^	25(20, 31)	23(18, 28)	27(21, 33)	<0.001
Immunoglobulin A (g/dL)^*^	0.330(0.253, 0.418)	0.336(0.253, 0.426)	0.326(0.252, 0.410)	0.580
Immunoglobulin G (g/dL)^*^	1.540(1.350, 1.760)	1.570(1.360, 1.820)	1.500(1.330, 1.690)	0.001
Immunoglobulin M (g/dL)^*^	0.106(0.075, 0.142)	0.100(0.072, 0.140)	0.109(0.081, 0.146)	0.012
Immunoglobulin E (IU/dL)^*^	0.272(0.091, 0.796)	0.364(0.110, 0.932)	0.222(0.082, 0.644)	<0.001
Immunoglobulin kappa (g/dL)^*^	0.400(0.339, 0.462)	0.418(0.359, 0.484)	0.379(0.325, 0.443)	<0.001
Immunoglobulin lambda (g/dL)^*^	0.205(0.178, 0.240)	0.206(0.178, 0.249)	0.205(0.178, 0.236)	0.242
Right atrium end-systolic diameter (mm)^*^	29(27, 32)	28(26, 31)	29(27, 32)	<0.001
Right ventricle end-diastolic diameter (mm)^*^	28(26, 31)	28(26, 31)	28(26, 30)	0.026
Pulmonary artery diameter (mm) ^*^	20(18, 21)	19(18, 21)	20(18, 21)	0.004
Left atrium end-systolic diameter (mm)^*^	30(27, 32)	29(26, 32)	30(27, 33)	0.001
Left ventricular end-diastolic diameter (mm)^*^	39(36, 42)	38(35, 41)	40(37, 43)	<0.001
Interventricular septum thickness (mm)^*^	10(9, 10)	9(8, 10)	10(9, 11)	0.003
Aorta diameter (mm)^*^	30(28, 32)	30(27, 32)	30(28, 33)	0.013
Left ventricular ejection fraction (%)^*^	60(58, 61)	60(58, 60)	60(58, 62)	0.062

^*^Median (interquartile range).

### Associations of abdominal fat and obesity, antibody and complement systems, and cardiac structure and function with centenarians

As shown in Table 2, PFT, WC, complement C3 level, complement C4 level, IgM level, RAESD, LAESD, and LVEDD were all inversely associated with centenarians (P<0.05 for all variables). A positive association with centenarians was instead detected for IgG level, immunoglobulin kappa level, and RVEDD (P<0.05 for all variables). In turn, no association with centenarians was found for IgA level, IgE level, immunoglobulin lambda level, PAD, IVST, AOD, and LVEF (P>0.05 for all variables).

**Table 2:** Characteristics associated with centenarians in logistic regression analysis.

**Characteristic**	**OR(95% CI)**	**P**
Periumbilical fat thickness (cm)	0.430(0.330-0.560)	<0.001
Waist circumference (cm)	0.965(0.948, 0.982)	<0.001
Complement C3 (mg/dL)	0.982(0.974, 0.989)	<0.001
Complement C4 (mg/dL)	0.962(0.945, 0.980)	<0.001
Immunoglobulin A (mg/dL)	1.000(0.999, 1.001)	0.467
Immunoglobulin G (mg/dL)	1.001(1.000, 1.001)	0.001
Immunoglobulin M (mg/dL)	0.997(0.995, 1.000)	0.032
Immunoglobulin E (IU/dL)	1.000(1.000, 1.000)	0.129
Immunoglobulin kappa (mg/dL)	1.004(1.003, 1.006)	<0.001
Immunoglobulin lambda (mg/dL)	1.002(1.000, 1.005)	0.082
Right atrium end-systolic diameter (mm)	0.941(0.906, 0.977)	0.001
Right ventricle end-diastolic diameter (mm)	1.058(1.015, 1.102)	0.008
Pulmonary artery diameter (mm)	0.957(0.902, 1.015)	0.140
Left atrium end-systolic diameter (mm)	0.960(0.930, 0.990)	0.010
Left ventricular end-diastolic diameter (mm)	0.893(0.864, 0.923)	<0.001
Interventricular septum thickness (mm)	0.908(0.821, 1.005)	0.061
Aorta diameter (mm)	0.973(0.937, 1.011)	0.160
Left ventricular ejection fraction (%)	0.980(0.942, 1.019)	0.314

Abbreviations: OR: odds ratio; 95%CI: 95% confidence interval.

Notes: With adjustment of age, sex, systolic blood pressure, diastolic blood pressure, fasting blood glucose, total cholesterol, triglyceride, high-density lipoprotein cholesterol, and low-density lipoprotein cholesterol.

### Associations of abdominal fat and obesity with antibody and complement systems and cardiac structure and function

As shown in Table 3, complement C3 level, RVEDD, PAD, LAESD, LVEDD, IVST, and AOD were all positively associated with both PFT and WC (P<0.05 for all variables). IgM and RAESD were inversely and positively associated, respectively, with WC (P<0.05) but not PFT (P>0.05). In contrast, no association with either PFT or WC was detected for complement C4 level, IgA level, IgG level, IgE level, immunoglobulin kappa level, immunoglobulin lambda level, and LVEF (P>0.05 for all variables).

**Table 3:** Associations of abdominal fat and obesity with antibody and complement systems and cardiac structure and function in linear regression analyses.

**Characteristic**	**Periumbilical fat thickness**		**Waist circumference**	
	**Standardized coefficient**	**P**	**Standardized coefficient**	**P**
Complement C3 (mg/dL)	0.067	0.041	0.151	<0.001
Complement C4 (mg/dL)	0.022	0.512	0.048	0.153
Immunoglobulin A (mg/dL)	-0.047	0.187	-0.029	0.424
Immunoglobulin G (mg/dL)	-0.052	0.146	-0.023	0.511
Immunoglobulin M (mg/dL)	-0.062	0.084	-0.074	0.037
Immunoglobulin E (IU/dL)	-0.021	0.550	-0.026	0.455
Immunoglobulin kappa (mg/dL)	-0.056	0.106	-0.036	0.302
Immunoglobulin lambda (mg/dL)	-0.032	0.368	0.031	0.391
Right atrium end-systolic diameter (mm)	0.059	0.089	0.082	0.017
Right ventricle end-diastolic diameter (mm)	0.156	<0.001	0.137	<0.001
Pulmonary artery diameter (mm)	0.140	<0.001	0.096	0.007
Left atrium end-systolic diameter (mm)	0.181	<0.001	0.271	<0.001
Left ventricular end-diastolic diameter (mm)	0.116	<0.001	0.164	<0.001
Interventricular septum thickness (mm)	0.186	<0.001	0.140	<0.001
Aorta diameter (mm)	0.145	<0.001	0.138	<0.001
Left ventricular ejection fraction (%)	0.056	0.120	-0.019	0.592

Notes: With adjustment of age, sex, systolic blood pressure, diastolic blood pressure, fasting blood glucose, total cholesterol, triglyceride, high-density lipoprotein cholesterol, and low-density lipoprotein cholesterol.

### Associations between antibody and complement systems and cardiac structure and function

As shown in Table 4, complement C3 level was inversely associated with RAESD, PAD, and IVST (P<0.05 for all variables). Complement C4 level was inversely associated with PAD (P<0.05). Immunoglobulin kappa level was positively associated with RAESD, LAESD, and LVEF (P<0.05 for all variables). Immunoglobulin lambda level was positively associated with RAESD and LAESD (P<0.05 for both variables). No association with cardiac structure and function was found for IgA, IgG, IgM, and IgE levels (P>0.05 for all variables).

**Table 4:** Associations of antibody and complement systems with cardiac structure and function in linear regression analyses.

**Characteristic**	**Right atrium end-systolic diameter (mm)**		**Right ventricle end-diastolic diameter (mm)**		**Pulmonary artery diameter (mm)**		**Left atrium end-systolic diameter (mm)**		**Left ventricular end-diastolic diameter (mm)**		**Interventricular septum thickness (mm)**		**Aorta diameter (mm)**		**Left ventricular ejection fraction (%)**	
	**Standardized coefficient**	**P**	**Standardized coefficient**	**P**	**Standardized coefficient**	**P**	**Standardized coefficient**	**P**	**Standardized coefficient**	**P**	**Standardized coefficient**	**P**	**Standardized coefficient**	**P**	**Standardized coefficients**	**P value**
Complement C3 (mg/dL)	-0.087	0.016	-0.032	0.376	-0.081	0.028	-0.059	0.098	-0.032	0.353	-0.073	0.045	-0.038	0.305	-0.065	0.086
Complement C4 (mg/dL)	-0.040	0.237	0.000	0.981	-0.077	0.028	-0.052	0.119	-0.059	0.069	-0.032	0.361	-0.061	0.078	-0.006	0.859
Immunoglobulin A (mg/dL)	0.055	0.090	-0.008	0.794	0.013	0.687	0.045	0.155	0.000	0.996	-0.042	0.196	0.016	0.638	-0.057	0.091
Immunoglobulin G (mg/dL)	0.020	0.548	0.023	0.487	-0.012	0.729	0.040	0.215	-0.022	0.472	0.003	0.924	0.005	0.877	-0.056	0.101
Immunoglobulin M (mg/dL)	0.025	0.422	0.026	0.431	0.003	0.929	0.052	0.100	0.020	0.519	0.000	0.997	-0.019	0.570	-0.030	0.377
Immunoglobulin E (IU/dL)	0.005	0.879	0.012	0.715	-0.008	0.797	0.038	0.225	0.031	0.306	-0.007	0.839	0.006	0.856	0.021	0.527
Immunoglobulin kappa (mg/dL)	0.077	0.020	0.060	0.072	0.005	0.872	0.094	0.004	-0.005	0.875	0.038	0.261	0.026	0.451	-0.110	0.001
Immunoglobulin lambda (mg/dL)	0.065	0.043	0.027	0.412	-0.029	0.376	0.075	0.018	-0.001	0.961	0.008	0.819	-0.034	0.297	-0.050	0.133

Notes: With adjustment of age, sex, waist circumference, systolic blood pressure, diastolic blood pressure, fasting blood glucose, total cholesterol, triglyceride, high-density lipoprotein cholesterol, and low-density lipoprotein cholesterol.

## DISCUSSION

We have witnessed over the last decades an alarming increase in abdominal obesity in virtually every country, irrespective of economic status and cultural background [1–4]. Our previous study indicated that abdominal obesity is associated with both arterial stiffness and impaired hemodynamics in Chinese adults [20]. Since abdominal obesity triggers chronic inflammation of adipose tissue and compromises immune function, we speculated that periumbilical fat may adversely influence longevity by altering the expression or the activity of the antibody-complement system. Although a few studies indicated an association between periumbilical fat and changes in antibody and complement profiles in Western populations [10, 11], it has not yet been determined whether such association impacts longevity, in either Western or Asian populations. The current study demonstrated that Chinese centenarians had less periumbilical fat and a weaker complement system compared with non-centenarian oldest-old population. Moreover, periumbilical fat was negatively associated with longevity mediated by complement C3 in the study’s total oldest-old population. These data indicate that abdominal obesity is an obstacle to successful aging and longevity in China.

Excess periumbilical fat can trigger innate immunity by producing cytokines that stimulate hepatic production of complement C3, an acute phase protein [21, 22]. Complement C3 is also synthesized by activated adipocytes and macrophages and functions as both a cytokine and an adipokine [23]. Complement C3 and other adipokines may promote insulin resistance (IR) by increasing phosphorylation and proteosomal degradation of insulin receptor substrates or by disturbing insulin receptor-substrate interaction [24]. As the main degradation product and active fragment of C3, acylation stimulating protein (ASP, C3a desArg) has insulin-like properties and favors lipid synthesis in adipocytes [25]. In the same way that IR determines increased insulin secretion, an increase in ASP precursor (C3) levels may be triggered by ASP resistance [26]. Since ASP is a potent stimulator of lipogenesis in adipocytes, increased complement C3 levels have the potential to disturb lipid metabolism and aggravate abdominal obesity [27]. Therefore, periumbilical fat may be partly responsible for increased mortality rate and reduced life expectancy mediated by complement C3 [28].

Substantial evidence indicates that metabolically abnormal obesity is associated with increased cardiovascular morbidity and mortality rates. Specifically, it has been proposed that abdominal obesity rather than overall obesity is linked to abnormal cardiac structure and function [29, 30]. Accordingly, a number of studies have confirmed the deleterious impact of obesity on life expectancy and longevity [9]. Still, scarce studies have been dedicated to analyzing the association between periumbilical fat and cardiac structure, and it remains unclear whether cardiac structure could mediate the association of periumbilical fat and longevity in either Western or Chinese oldest-old populations [13]. The current study showed that Chinese centenarians had less periumbilical fat and smaller cardiac structure compared with non-centenarian oldest-old population. In turn, periumbilical fat was negatively associated with longevity mediated by cardiac structure in the study’s total oldest-old population. There are several reasons why periumbilical fat might affect cardiac structure and reduce life expectancy. They include cardiac effects related to IR, which can be partly driven by periumbilical fat via increased fatty acid secretion, decreased adiponectin secretion, and promoted hyperinsulinemia development [31]. In turn, cardiac effects of hyperinsulinemia, including myocardial hypertrophy, can result among other factors from disturbed electrolyte balance, oxidative stress, enhanced sympathetic tone, and dysfunction of the renin-angiotensin-aldosterone system [32]. Excess periumbilical fat may also contribute to the development of an abnormal cardiac structure by releasing proinflammatory cytokines. These effects may be further compounded by genetic and epigenetic associations between periumbilical fat and cardiac structure [33].

The effects of immune activation on cardiac structure are time-dependent. After an acute injury event, like myocardial infarction, the innate immune system is activated as a prerequisite for adequate healing [34]. However, long-term innate immune activation is detrimental, resulting in adverse cardiac remodeling and structural and functional compromise of the organ [35]. C3 seems to be not only a good indicator of overall complement activation but may also be of pathophysiological relevance in the cardiovascular system. Experimental evidence from animal studies suggests that complement activation is mechanistically involved in adverse cardiac healing and remodeling [36]. For instance, it was shown that elevated C3 levels lead to abnormal left ventricular structure and contractile failure in isolated guinea pig hearts [37]. C3 is an effector protein that can efficiently attract inflammatory cells and directly destroy cells by the complement membrane attack complex. To our knowledge, this is the first study detecting an inverse association between complement C3 and cardiac structure in Chinese oldest-old population, including centenarians.

## CONCLUSIONS

Based on CHCCS data, the current study demonstrated a complex association network between periumbilical fat and longevity mediated by complement C3 and cardiac structure in Chinese oldest-old population, including centenarians. By highlighting the associations of both complement C3 and cardiac structure with centenarian status, our study suggests that preventing abdominal obesity represents a key strategy to promote successful aging. Nevertheless, molecular studies are required to explore the specific mechanisms underlying the associations identified in the current study.

## MATERIALS AND METHODS

### Study population

The population-based CHCCS was carried out in 18 cities and counties of Hainan Province, China. It started in July 2014 and ended in December 2016, and its cohort profile has been described previously [38]. There were 910 oldest-old participants (at least 80 years of age) identified from the National Civil Registry from Hainan Civil Affairs Bureau. Age was ascertained from national identification cards. The current study was carried out after approval of the Ethics Committee of Hainan Hospital of Chinese People’s Liberation Army General Hospital (Sanya, Hainan; Number: 301hn11201601). All participants provided written informed consent prior to the start of the study.

### Standard procedures

Based on a standardized protocol, face interviews, physical examinations, and blood analyses were administered by a well-trained research team from the Chinese People’s Liberation Army General Hospital using a home-visiting model. This interdisciplinary research team included internists, geriatricians, cardiologists, endocrinologists, nephrologists, and nurses. Based on the recommendations of the World Health Organization, waist circumference (WC) was measured in standing subjects using a flexible tape, midway between the lowest rib and the iliac crest. Blood pressure was measured on the participants’ right arms with a calibrated desktop sphygmomanometer (Yuwell medical equipment and supply Co., Ltd., Jiangsu, China). Appropriate cuff sizes were determined based on arm circumference. Participants sat in a chair for five minutes with their feet on the floor and the right arm supported at heart level. Systolic blood pressure (SBP) and diastolic blood pressure (DBP) were recorded at the first and fifth Korotkoff sounds, respectively, and reported as the average of two separate measurements at an interval of more than one minute.

Samples of venous blood were routinely drawn by venipuncture, stored at 4° C, and delivered within four hours to the central laboratory at the Department of Biochemistry, Hainan Hospital of Chinese People’s Liberation Army General Hospital. Serum levels of immunoglobulins and complement components were measured with a fully automatic protein analyzer (BNII; Siemens AG, Munich, Germany). Serum levels of triglyceride (TG), total cholesterol (TC), high-density lipoprotein cholesterol (HDL-C), and fasting blood glucose (FBG) were measured by enzymatic assays (Roche Products Ltd, Basel, Switzerland) using a biochemical autoanalyzer (Cobas c702; Roche Products Ltd), with low-density lipoprotein cholesterol (LDL-C) calculated by the Friedewald equation. Qualified technicians who performed laboratory analyses were blinded to clinical data.

### Ultrasound examinations

A portable ultrasound machine (Philips CX50, Philips Medical Systems, Andover, MA, USA) was used to measure periumbilical fat thickness (PFT) and parameters of cardiac structure and function by experienced radiologists who were unaware of clinical and laboratory data. All participants were placed in a supine position and the abdomen was scanned using a 3-12 MHz linear array ultrasound transducer (L12-3; Phillips). Cardiac ultrasound was performed using a 1-5 MHz cardiac transducer (S5-1; Phillips).

### Statistical analyses

Continuous variables were described using means with standard deviations for data with normal distribution, and medians with interquartile ranges for data with skewed distribution. Categorical variables were described as numbers and percentages. Comparisons between groups were made using Student’s *t*-test for continuous variables with normal distribution, Mann–Whitney *U* test for continuous variables with skewed distribution, and *χ^2^* test for categorical variables. Logistic regression was performed with centenarians as dependent variable, adjusting for age, sex, SBP, DBP, FBG, TC, TG, HDL-C, and LDL-C. PFT, WC, and parameters of cardiac structure were logarithmically transformed to meet the multinormality assumption. Linear regression was performed with PFT, WC, and parameters of cardiac structure as dependent variables, adjusting for age, sex, SBP, DBP, FBG, TC, TG, HDL-C, and LDL-C. Age and sex are demographic characteristics, whereas SBP, DBP, FBG, TC, TG, HDL-C, and LDL-C are generally believed to be related to PFT, WC, and risk factors for cardiac structure. Statistical analyses were made with SPSS version 17 (IBM Corporation, Armonk, NY, USA) at the significance level of P<0.05 for two-sided tests.
